# Phylotastic! Making tree-of-life knowledge accessible, reusable and convenient

**DOI:** 10.1186/1471-2105-14-158

**Published:** 2013-05-13

**Authors:** Arlin Stoltzfus, Hilmar Lapp, Naim Matasci, Helena Deus, Brian Sidlauskas, Christian M Zmasek, Gaurav Vaidya, Enrico Pontelli, Karen Cranston, Rutger Vos, Campbell O Webb, Luke J Harmon, Megan Pirrung, Brian O'Meara, Matthew W Pennell, Siavash Mirarab, Michael S Rosenberg, James P Balhoff, Holly M Bik, Tracy A Heath, Peter E Midford, Joseph W Brown, Emily Jane McTavish, Jeet Sukumaran, Mark Westneat, Michael E Alfaro, Aaron Steele, Greg Jordan

**Affiliations:** 1 Institute for Bioscience and Biotechnology Research (IBBR), Biosystems and Biomaterials Division, National Institute of Standards and Technology, Gaithersburg, MD, 20899, USA; 2 National Evolutionary Synthesis Center, 2024 W. Main St, Durham, NC, 27705, USA; 3 The iPlant Collaborative and EEB Department, University of Arizona, 1657 E Helen St, Tucson, AZ, 85721, USA; 4 Digital Enterprise Research Institute, National University of Ireland, University Road, Galway, Ireland; 5 Department of Fisheries and Wildlife, Oregon State University, 104 Nash Hall, Corvallis, OR, 97331-3803, USA; 6 Sanford-Burnham Medical Research Institute, 10901 North Torrey Pines Road, La Jolla, CA, 92037, USA; 7 Department of Ecology and Evolutionary Biology, University of Colorado Boulder, Boulder, CO, 80309-0334, USA; 8 Department of Computer Science, New Mexico State University, MSC CS, Box 30001, Las Cruces, NM, 88003, USA; 9 NCB Naturalis, Einsteinweg 2, Leiden, 2333 CC, the Netherlands; 10 Arnold Arboretum of Harvard University, Boston, MA, 02130, USA; 11 Institute for Bioinformatics and Evolutionary Studies (IBEST), University of Idaho, PO Box 443051, Moscow, ID, 83844-3051, USA; 12 University of Colorado Denver Anschutz Medical Campus, Aurora, CO, 80045, USA; 13 Department of Ecology & Evolutionary Biology, 569 Dabney Hall, University of Tennessee, Knoxville, TN, 37996, USA; 14 Department of Computer Science, University of Texas at Austin, Austin, TX, 78701, USA; 15 Center for Evolutionary Medicine and Informatics, The Biodesign Institute, and School of Life Sciences, Arizona State University, PO Box 874501, Tempe, AZ, 85287-4501, USA; 16 UC Davis Genome Center, One Shields Ave, Davis, CA, 95618, USA; 17 Department of Integrative Biology, University of California, Berkeley, CA, 94720-3140, USA; 18 University of Texas at Austin, BEACON, Austin, TX, USA; 19 Biology Department, Duke University, Biological Sciences Building, 125 Science Drive, Durham, NC, 27708, USA; 20 Biodiversity Synthesis Center, Field Museum of Natural History, 1400 S Lakeshore Dr, Chicago, IL, 60605, USA; 21 Department of Ecology and Evolutionary Biology, South University of California Los Angeles, 621 Charles E. Young Dr, Los Angeles, CA, 90095, USA; 22 U.C. Berkeley Museum of Vertebrate Zoology, University of California, 3101 Valley Life Sciences Building, Berkeley, CA, 94720, USA; 23 Paperpile, 34 Houghton Street, Somerville, MA, 02143, USA

**Keywords:** Phylogeny, Taxonomy, Hackathon, Web services, Data reuse, Tree of life

## Abstract

**Background:**

Scientists rarely reuse expert knowledge of phylogeny, in spite of years of effort to assemble a great “Tree of Life” (ToL). A notable exception involves the use of *Phylomatic*, which provides tools to generate custom phylogenies from a large, pre-computed, expert phylogeny of plant taxa. This suggests great potential for a more generalized system that, starting with a query consisting of a list of any known species, would rectify non-standard names, identify expert phylogenies containing the implicated taxa, prune away unneeded parts, and supply branch lengths and annotations, resulting in a custom phylogeny suited to the user’s needs. Such a system could become a sustainable community resource if implemented as a distributed system of loosely coupled parts that interact through clearly defined interfaces.

**Results:**

With the aim of building such a *“phylotastic”* system, the NESCent *Hackathons, Interoperability, Phylogenies (HIP)* working group recruited 2 dozen scientist-programmers to a weeklong programming hackathon in June 2012. During the hackathon (and a three-month follow-up period), 5 teams produced designs, implementations, documentation, presentations, and tests including: (1) a generalized scheme for integrating components; (2) proof-of-concept pruners and controllers; (3) a meta-API for taxonomic name resolution services; (4) a system for storing, finding, and retrieving phylogenies using semantic web technologies for data exchange, storage, and querying; (5) an innovative new service, *DateLife.org*, which synthesizes pre-computed, time-calibrated phylogenies to assign ages to nodes; and (6) demonstration projects. These outcomes are accessible via a public code repository (GitHub.com), a website (http://www.phylotastic.org), and a server image.

**Conclusions:**

Approximately 9 person-months of effort (centered on a software development hackathon) resulted in the design and implementation of proof-of-concept software for 4 core phylotastic components, 3 controllers, and 3 end-user demonstration tools. While these products have substantial limitations, they suggest considerable potential for a distributed system that makes phylogenetic knowledge readily accessible in computable form. Widespread use of phylotastic systems will create an electronic marketplace for sharing phylogenetic knowledge that will spur innovation in other areas of the ToL enterprise, such as annotation of sources and methods and third-party methods of quality assessment.

## Background

Researchers in many areas of life sciences, from community ecology to genomics to biomedical genetics, use phylogenies to place data in an evolutionary context [1]. Phylogenies provide the basis for classification, whether of species (i.e., biological taxonomy) or molecular sequences. Furthermore, phylogenies are central to rigorous quantitative methods of comparative analysis used throughout biology. Evolved things (genes, species, or other entities) have features that are highly correlated by virtue of common ancestry, thus they are not independent samples of an underlying process, but require special methods of analysis: evolutionary comparative methods use branching models to separate correlations due to common ancestry from correlations due to functional causes.

Inferring a phylogeny is often a challenging task with multiple steps, subject to numerous pitfalls [2]. To infer a credible tree, users must collaborate with experts, or commit to learning about phylogenetic methods.

Nevertheless, the number of phylogeny publications has been growing at a rate considerably above the baseline growth of scientific publishing (e.g., compare [3] with [4]). In 2010, an estimated 7700 publications reported new phylogenies [5]. These and other phylogenies computed throughout the life sciences collectively represent the sum of expert knowledge of evolutionary relationships. This knowledge is largely scattered and inaccessible, locked inside individual publications. In spite of a community archive that has existed for many years (TreeBASE [6,7]), roughly 96% of phylogenies are not archived, and are available only as pictures in a scientific journal [5].

One possible interpretation of this situation is that, in spite of the effort that goes into generating phylogenies, they generally have a very low value for re-use. One might argue that phylogenies are volatile and must be re-computed constantly from an ever-expanding body of data using ever-improving methods. If so, then the lack of archiving and re-use of phylogenies is neither surprising nor problematic.

However, a recent study of phylogeny re-use [5] suggests that certain types of phylogenies have a high re-use value, under the right conditions. In a small sample of just 40 phylogeny-relevant research articles, the authors found that 6 of the studies re-used large trees, 4 of them using the software called *Phylomatic*[8] to perform pruning and grafting operations on the framework tree provided by the Angiosperm Phylogeny Group (APG). The APG tree [9] aims to cover all flowering plants, albeit mostly at the level of higher taxa (e.g., families, orders) rather than of species. In this context, “pruning” means cutting away unwanted terminal nodes (and collapsing any resulting unnecessary internal nodes), while “grafting” means adding branches to a tree, which Phylomatic does taxonomically (i.e., “taxonomic grafting”, based on an input list with taxonomic derivations of the form “<order>/<family>/<genus>/<genus species>”). Phylomatic has been cited in over 200 scientific articles since 2005 [10].

In other words, Phylomatic uses simple manipulations to generate a customized tree based on a larger authoritative tree computed by experts, providing the user with a combination of convenience and credibility. The expert trees most useful in such cases will be those that **(1)** address the relationships of species (as opposed to relationships of genes or proteins) and **(2)** cover a large number of species. A single ToL covering millions of species does not exist (in spite of a decade-long effort by the US National Science Foundation), but there are many trees that provide extensive coverage of a large group, e.g., 4510 extant mammal species [11], 55473 angiosperms [12], 73060 eukaryotic species [13], and 408135 prokaryotic 16S rDNAs in the “greengenes” tree [14]. To this list, one may add resources that are not true phylogenies, but taxonomic hierarchies, including the NCBI taxonomy hierarchy of 250000 species of prokaryotes, eukaryotes, and viruses [15], the downloadable version of the tree from the Tree of Life Web project [16] with 16000 species, and the APG tree with 1566 taxa [9]. The NCBI hierarchy is widely used as a ToL in projects that require a single unifying framework to cover all domains of life (e.g., [17-21]).

Whereas the lack of a single authoritative ToL may be a substantial barrier to the re-use of expert knowledge of species phylogeny, the lack of a convenient **delivery system** for available ToL knowledge is a barrier of equal or greater importance. In particular, the example of Phylomatic suggests the potential for a more general system that, in response to a query consisting of a list of species (or higher taxa), rapidly supplies a phylogeny for those species based on expert knowledge. Ideally such a system would cover the entire ToL, be fast enough to provide results while the user waits, and address the particular needs of researchers for reproducibility and provenance. Such a system will not replace the time-consuming generation of robust phylogenies by experts, but aims to make those hard-won results conveniently accessible to everyone else.

In this paper, we report initial results of a project, codenamed “phylotastic”*,* that aims to create such a system. The HIP (Hackathons, Interoperability, Phylogenies) working group of the National Evolutionary Synthesis Center (NESCent) gathered a group of programmer-scientists for a one-week “hackathon” (an intensive bout of collaborative software development) aimed at building a system of loosely coupled components that collectively provide phylotastic access to phylogenies with broad taxonomic coverage. The system was designed and implemented by 5 teams: the Architecture team was responsible for overall design and controllers; the TNRS team focused on *Taxonomic Name Resolution Services*; the TreeStore team developed methods to submit, store, and retrieve phylogenetic trees; the Branch Lengths team implemented a system to assign divergence times to nodes; and team Shiny focused on end-user experiences and outreach. Project outcomes are accessible via a public code repository (http://github.com/phylotastic), a project web site (http://www.phylotastic.org), and a server image (supplementary data).

Our results show that providing on-the-fly phylogenies via web services is possible, although improvements are needed in order to create a robust and flexible system that meets the typical demands of researchers. With further development, phylotastic systems have the potential to create an electronic marketplace for sharing phylogenetic knowledge that, in turn, may be expected to provide the incentives for researchers and technologists to improve data quality, improve technology and standards for annotation of sources and methods, and facilitate third-party methods of quality assessment.

## Implementation

### Hackathon planning and execution

The goal of developing a phylotastic system emerged in January 2012 from brainstorming and evaluation of multiple alternatives at a face-to-face meeting of the HIP leadership team (LT), a group of 10 scientists with backgrounds in molecular biology, bioinformatics, molecular evolution, genomics, phylogenetics, and comparative biology. Hackathon participants were selected from applications submitted in response to an open call for participation. From 29 applications—nearly all from qualified individuals—the LT selected 20 participants to represent a breadth of expertise and knowledge. A simultaneous satellite hackathon was arranged with a remote group of 6 individuals.

In the two-month pre-hackathon planning stage (April to May of 2012), participants were enrolled in a mailing list and encouraged to raise issues and share ideas. LT members regularly injected ideas and challenges to stimulate discussion and maintain energy and momentum. Some participants and organizers developed and shared proof-of-concept software during this period. By the time of the hackathon, nearly all participants had engaged in discussions via telephone conferencing and a shared email list.

The hackathon took place from June 4 to 8, 2012 at NESCent headquarters in Durham, NC, with a satellite hackathon in Moscow, Idaho composed of a group of interested researchers who could not travel to Durham. The first day began with user scenarios (descriptions of how researchers use ToL knowledge); a two-hour brainstorming session; short technical talks to familiarize participants with certain key technologies (NeXML, the semantic web, iPlant TNRS); and an open-space session to form teams. During the open-space session, participants proposed, joined, critiqued, revised and defended “pitches” (proposals) in an open room, until unpopular pitches were abandoned and the group settled down to a limited number of teams. The first day ended with 5 teams, and the remaining 3.5 days were spent planning and executing team projects. In the period after the hackathon, organizers and a subset of participants collaborated remotely to improve hackathon products.

### General conception of a phylotastic system

In the pre-hackathon stage, and during the first day, participants developed a generalized view of phylotastic systems as a means to deliver ToL knowledge to researchers. This view is intended to be practical given present realities of the informatics landscape, yet extensible and adaptable as a long-term community resource.

#### Conditions

There are 2 initial conditions of vital importance. The first is that the user has a list of taxa, typically a list of dozens or hundreds of species. Rarely the list may be longer; it may contain higher taxa such as genera, families or orders. The input list might be composed manually by the user, or constructed interactively from some data resource, e.g., the user might invoke the Global Names Recognition & Discovery service (see Table 1) on the PDF of a scientific paper, resulting in the list of taxa named in that document. The lists of names that emerge from ordinary scientific sources frequently have errors in the specification of names, including typographical errors, misspellings based on ignorance, and deprecated names, with the result that integrating data using names as keys is a major bioinformatics challenge [22-24].

**Table 1:** Locations of online resources, with hackathon team listed where appropriate

**Team**	**Description of resource**	**URI**
-	Forester Project: software libraries for evolutionary biology and comparative genomics research	https://code.google.com/p/forester/
-	GNRD: Global Names Recognition and Discovery	http://gnrd.globalnames.org
-	ITIS: Integrated Taxonomic Information System	http://www.itis.gov
-	Mesquite: A Modular System for Evolutionary Analysis	http://www.mesquiteproject.org
-	MSW3: Mammal Species of the World	http://www.bucknell.edu/msw3/
-	NCBI Entrez Programming Utilities (E-Utilities)	http://www.ncbi.nlm.nih.gov/books/NBK25500/
-	Open Tree of Life project	http://opentreeoflife.org
-	UniProt Web services	http://www.uniprot.org/faq/28
All	Phylotastic Main Web Site	http://www.phylotastic.org
All	Phylotastic Server Image	http://nescent.org/download/phylotastic.ova
Arch	Phylotastic Galaxy Instance	http://galaxy.phylotastic.org
Arch	Using Phylotastic Tools Inside Galaxy (Screencast)	http://www.youtube.com/watch?v=kMME658xOu4
Arch	Phylotastic MapReduce-based Tree Pruner	http://phylotastic-wg.nescent.org/script/phylotastic.cgi
Arch	Phylomatic Version 3	http://phylodiversity.net/phylomatic/
Branch	DateLife: When Lineages Meet (Presentation)	http://www.youtube.com/watch?v=xWmLrbUWtyM
Branch	DateLife Project	http://www.datelife.org
Shiny	Mesquite-o-Tastic screencast	http://www.youtube.com/watch?v=fdMQOVdQ8eM
TNRS	API description for TNRS	http://www.evoio.org/wiki/Phylotastic/TNRS
TNRS	Phylotastic TNRS service	http://api.phylotastic.org/tnrs
TreeStore	Phylotastic Taxonomic Name Resolution Ontology	http://phylotastic.org/terms/tnrs.rdf

In case of difficulty resolving a URI to the expected resource, contact the authors or consult the online version of this table (http://www.phylotastic.org/docs/pub1table1.html).

The second important initial condition is that there are a variety of sources of expert phylogenetic knowledge, in the form of multiple source trees that might satisfy (partially or fully) the user’s query (examples were listed in Background). There is no single authority for such trees. They are available in many formats, although this is not problematic due to the availability of general-purpose libraries that provide tools for format conversion [25,26]. In some cases, the trees that are most useful are taxonomic hierarchies with polytomies, missing branch lengths, and higher taxa as terminals (e.g., [9]). While truly global phylogenies may appear very soon, we assume that, for years into the future, expert phylogenetic knowledge will reside in a plurality of trees, not a single tree.

#### Requirements

The basic functional requirement is to provide a phylogeny in response to the user’s query, which consists of a list of entities (typically, a list of species) and optionally, additional conditions (e.g., specifying a particular source tree, a restriction on name-matching, etc.). One may conceive of the user’s query as an under-specified version of the resulting phylogeny, i.e., a graph of unconnected leaf nodes that is filled in by the remaining components of the system.

Importantly, the utility of such a system depends on its strengths and weaknesses relative to the alternative (faced by the user) of (1) obtaining specialized training in phylogenetic inference methods, (2) installing or otherwise accessing specialized software, and (3) executing a workflow to infer a custom phylogeny from character data— all of which require a considerable expenditure of time to produce a tree that nonetheless may lack credibility. In the system envisioned here, phylogenetic knowledge is returned while the user waits, and ultimately comes from phylogenies produced by experts and presented (typically) in stand-alone publications (e.g., [11]).

Currently, many such source trees (e.g., [9,16]) include polytomies and lack branch lengths, yet users typically require a fully resolved tree that includes branch lengths. The reason for this is that branch lengths are required to apply various downstream (phylogeny-based) methods, such as independent contrasts [27], probabilistic reconstruction of ancestral states [28], or correlation of discrete traits [29]. Many implementations of these methods require fully resolved trees (i.e., trees without polytomies), even if the method itself does not. Branch lengths may be obtained in units of amount of change by using tree inference techniques with character data (e.g., sequences from GenBank). Inclusion of calibrations or constraints from fossils, biogeographical data, or other sources can be used with a tree to infer branch lengths in time units. A method called bladj, included in the Phylomatic package [8], simply assigns lengths so that branchings are evenly spaced.

Furthermore, it should be possible to cite the derived tree and provide a description of provenance in a way that will satisfy expectations for writing the “Methods” section of a scientific publication. Such a description would identify the source tree, describe the manipulations performed on it, and possibly provide a precise means to recover the derived tree.

In this context, one may imagine a system that takes the user’s query, rectifies names, identifies a source tree with the best coverage of the user’s list of species, invokes pruning and grafting operations to provide a phylogeny for the species, and as needed, invokes further services to provide branch lengths and possibly other annotations. The name-rectifier would accept, as input, a list of names, and would return a mapping of the input names to qualified taxonomic identifiers. Tree-stores would accept, as input, a query with descriptors or conditions to be satisfied (coverage, quality), and would return trees (or references to trees) that satisfy the conditions. Pruners (and grafters) would accept (1) a list of names and (2) a tree (or tree reference), and would return a suitably pruned (or grafted) tree. Alternatively, one may imagine topology services that combine information from multiple trees (so-called supertree methods) rather than simple pruning and grafting. Scaling operations would accept, as input, a tree with or without branch lengths, and would return a tree with newly scaled branch lengths, or with dates assigned to nodes.

Such a system would be useful to a wide variety of users. Because most such users are not computing experts, one cannot expect them to navigate the complex series of operations just described. Instead, we assume that most users will take advantage of phylotastic systems via client applications or controllers that manage a phylotastic workflow. This raises the question of how to design the overall architecture of the system. We assume that a system that is distributed and based on open collaboratively developed standards has a greater likelihood of becoming a sustainable community resource, relative to a closed, centralized system. Openness and collaborative development lower the bar to participation by early adopters and increase the breadth of use-cases being served, and thus are considered important factors favoring sustainability of cyberinfrastructure [30,31].

**Figure 1:**
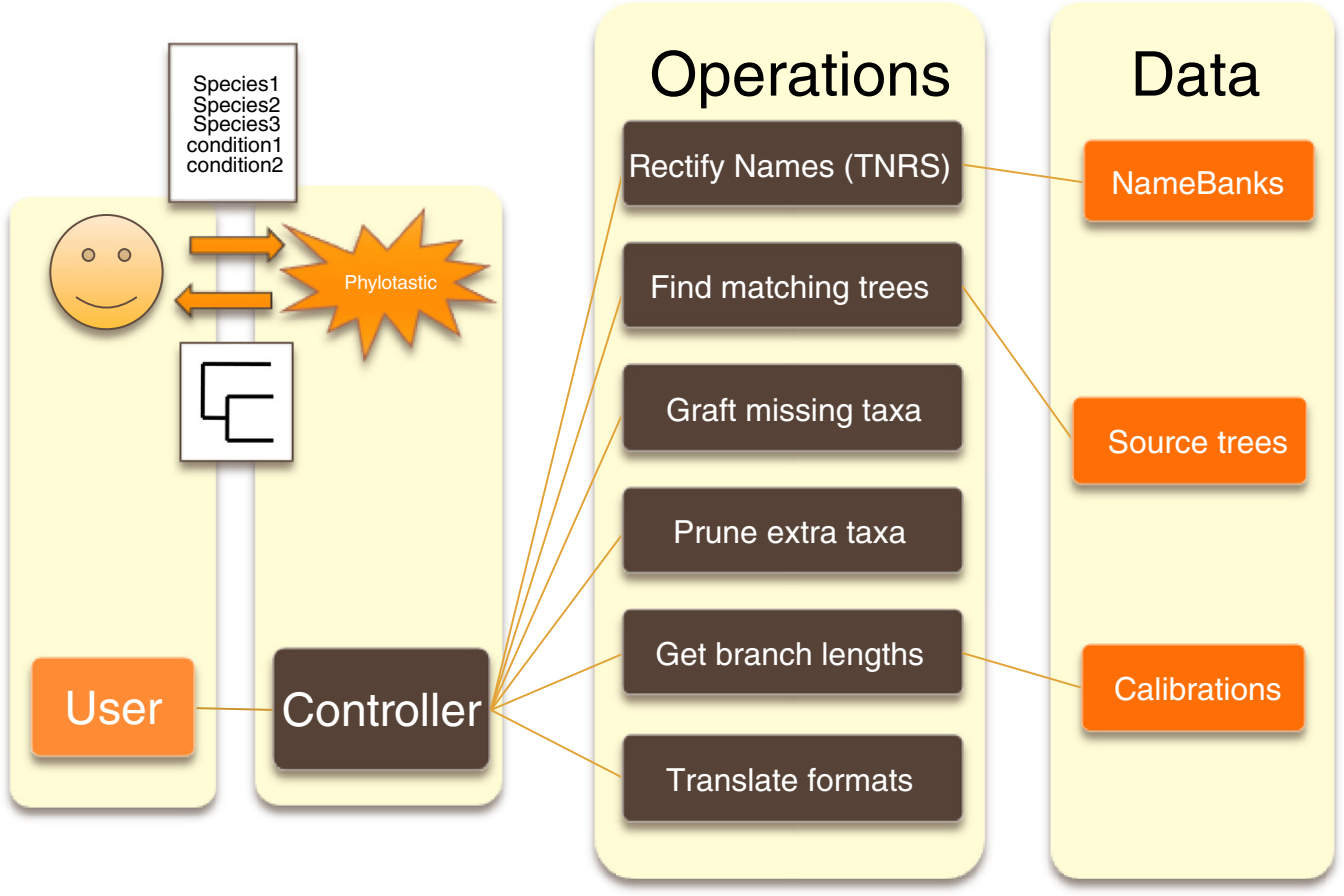
**Overall scheme of a phylotastic system.** The user (upper left) experiences a phylotastic system as a piece of software that returns a phylogeny in response to a query consisting of a list of taxa (and possibly other qualifiers). The user’s point of access to the system is a client program or controller that invokes various operations to access (and transform) the information needed to satisfy the user’s query. The response ultimately depends on information available from name-banks, source trees, and calibrations (right). There are many ways to implement such a system. In the approach described here, it is a system of loosely coupled components that uses web services to exchange information using common standards.

Therefore, to facilitate the development and maintenance of phylotastic systems as a sustainable community resource, we imagine the system in Figure 1 as a set of loosely coupled web services. That is, we imagine a step such as taxonomic name resolution, not as a local operation—e.g., based on a local library of taxonomy-related functions that access a local namebank—but as a remote web service maintained by taxonomy experts (e.g., the ITIS service: see Table 1). Furthermore, we imagine that, for each type of service, there are many possibilities, rather than a single authoritative service. Thus, one phylotastic client might access TNRS service #1. and another client might access TNRS service #2. This feature will allow the possibility for phylotastic systems to be maintained, without relying on the continuity of any particular service or resource. Ultimately, different scientific groups may expose their preferred phylogenies, TNRSs, pruners, and so on; likewise, different clients ultimately would be able to choose among multiple services based on quality, reliability, coverage, and so on.

Finally, given multiple services of each type, one can imagine each operation in Figure 1, not as a single instance of a resource, but as a service broker that accesses a registry of many services, invoking whichever service is most appropriate to process the user’s query, as in the BioMoby registry [32]. Furthermore, these brokers might choose services using indicators of quality or reliability, based on the success of past queries, or based on third-party evaluations (e.g., social bookmarking).

### Architecture

The architecture team focused on the high-level design of phylotastic systems, including the definitions and interaction of components, the flow of information through standard interfaces, and the integrated control of operations via controllers and workflow environments.

#### Design considerations

The architecture group set out to identify and formalize the interoperation between different components described in the generalized design above, taking into account (1) agreement on the minimum workflow to be enabled; (2) a delimitation of operations into modules with agreed inputs and outputs, in terms of both format and content; (3) the requirement that all modules can operate both standalone and as part of a workflow; and (4) the requirement that the system is driven by user events. Addressing these requirements ensured that each individual module could be developed independently and treated as a “black box” in the overall architecture, taking input in a specific format, and producing output to be reused by other modules.

The minimum functionality requirement devised by the hackathon participants was the following: when a user submits a list of names through a controller interface, an annotated tree containing all the named species (“phylotastic tree”) is returned. To enable this simple workflow, the submission of names (“dirty names”) to the controller triggers the TNRS module, which makes use of public services to return a new list of names (“clean names”). The controller submits the clean names to a tree-store service, which will return the matching megatrees and associated metadata. The topology service, in turn, finds and retrieves applicable megatrees by querying the tree-store. Optionally, the user may pass (as input to the topology service) reference trees to be used by the topology service. Both megatrees (returned by the tree-store) and phylotastic trees (pruned and returned by the topology service) can be enriched by the annotator, which tags each branch of the selected tree with provenance metadata and branch length. The phylotastic trees are returned to the user through a user interface that may enable the user to visualize and manipulate the tree. Note that inputs and outputs may be passed by reference (e.g., components may pass a megatree via a resolvable reference to the megatree).

#### Implementations

**Figure 2:**
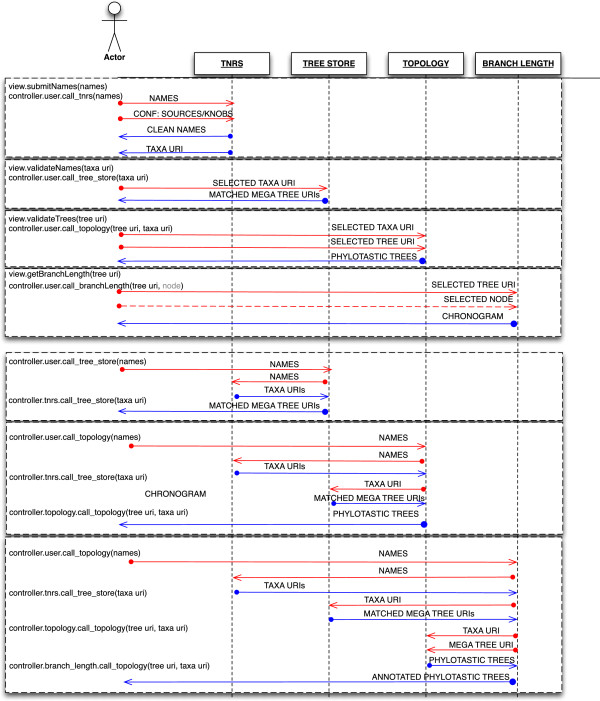
**Use case diagram for the implementation of the reference architecture, displaying both the minimal workflow requirements (upper) and the complete workflow (lower) with module independence.** The method names are represented in the left column. When a list of names is submitted to the controller, the TNRS endpoint is invoked with a set of configuration parameters. This list of URI names is then submitted to the tree-store service and the output expected is a set of matched megatrees. The megatree URIs and the user-selected taxa URIs are then submitted to the topology endpoint, which responds with the phylotastic trees. Finally, the URIs are submitted to the Branch Length module, which returns the chronogram or trees annotated with branch lengths.

Since one of the requirements was that all services were able to interoperate, whereas the hackathon teams used a variety of programming paradigms, the agreed format and protocol for exchanging data was through REST services. Figure 2 shows the flow of operations for a typical use-case, as the user’s query is processed via various components of a phylotastic system.

To demonstrate the platform-independent and loosely coupled nature of the phylotastic scheme, 3 different controllers were implemented, in JavaScript, Perl and the component API for Galaxy [33], a workflow environment. The JavaScript controller (executable on the server side with Node.js [34], or in the client side through any web browser) was designed with 4 endpoints to match each of the independently developed modules: TNRS, Tree Store, Topology and Branch Length (Figure 2). The controller both supports the minimum use case, and respects the independence requirement of each module. The Perl version of the controller is implemented as a CGI script and provides functionality that is similar to the JavaScript controller. By default, the Perl controller coordinates 4 “dummy” implementations of the TNRS, Tree Store, Topology, and Branch Length modules, but can be configured (via CGI parameters) to invoke real implementations for each of these modules.

Finally, a controller was implemented in the form of a collection of simple clients for the Galaxy platform [33]. Using Galaxy’s interactive workflow editor, these clients can be chained together in flexible ways to perform taxonomic name reconciliation, branch length estimation and tree pruning using the previously described RESTful services, in addition to various file conversion and filtering services to accomodate the tabular data model used in the Galaxy environment.

### Resolving taxonomic names (TNRS)

The phylotastic system envisioned above integrates data using species names, but errors and lack of specificity in such names can be major sources of ambiguity [22,35]. Users attempting to integrate phylogenetic information via names must go through a manual process of reconciling these names to each other. Several TNRSs (taxonomic name resolution services) have been created in recent years that may assist in this process by matching user-supplied names against currently valid or accepted names in taxonomic databases (e.g., ITIS: see Table 1). However, each TNRS service uses a different API, has a different number of names in its database, covers different sections of the ToL and provides a different set of features [36]; none represents a complete solution for a phylotastic system. We decided to focus on developing a single meta-service that would provide access to multiple existing TNRSs, as well as a single API, which could in the future be used by any client software to query any TNRS.

#### Design considerations

Our main design goals were to develop a web service and interface which would be (1) easy for developers to integrate into phylotastic workflows, (2) simple enough for users to understand, but without shielding the complexity of taxon names from them, and (3) broad enough to cover multiple nomenclatural codes. With these aims in mind, we developed a dual-purpose API that serves both for core services and for a meta-service that aggregates over multiple core services.

The API, which is documented more fully on the project wiki linked to the project web site (Table 1), is based on the iPlant TNRS API [36], and always returns responses as correctly formatted JavaScript Object Notation (JSON) objects. It provides only two methods: a *submit* method which accepts a list of newline-separated names for matching, returning a token, and a *retrieve* method that accepts a token and returns a report on the results of processing the original query. This asynchronous approach allows the server to carry out computationally intensive processes like fuzzy matching without the risk of the TCP/IP connection timing out.

For each name submitted, every name matched by every service is returned with a match score between 0.0 and 1.0, with 0 indicating that the name could not be matched and 1 indicating a perfect match. This score can be used by TNRSs that implement fuzzy or partial matching algorithms to report match scores or degrees of certainty. The meta-service returns all names found across all queried sources, leaving it to the client to decide which names to pick in case of conflicts between sources. A URI uniquely identifies each accepted name and provides credit to its source. Details are available in the online API description mentioned above.

#### Implementations

The reference implementation (codenamed “Taxosaurus”) consists of a handler and a collection of adaptors. The handler is responsible for communicating with the client and the adaptors; it uses a subset of the full API to send the client's queries to each adaptor, and combines and formats the results from each adaptor. The calls to the name-providers are handled by adaptors that are independent from the handler, and which may be written in any programming language. Their task is to serve as a translator between the meta-service API and the name provider’s API. The handler itself is modular; for purposes of speed, parts of it might eventually be incorporated into the Apache web service. Taxosaurus is very similar to the Taxonomic Search Engine of Page [35]. A key difference is the adoption of a RESTful approach instead of SOAP and the use of URIs instead of LSIDs to uniquely identify each accepted name.

A core service that conforms to the API may be aggregated into the meta-service implementation; a TNRS service that does not conform to the API may be wrapped in an adaptor, in principle. We provide access to three core services described below.

*NCBI Taxonomy*. This adaptor, written in Python, uses NCBI’s E-Utils service (particularly the “ESearch” and “ESummary” commands) to match names (see Table 1 for NCBI eUtils). Scores may be '1' (successful match) or '0' (could not match).

*iPlant TNRS*. Since our API design was directly inspired by the iPlant API [36], this adaptor consists of a simple Perl script that forwards queries to iPlant, and renames field names in their result to our schema before returning the results to the handler. The score returned by the iPlant TNRS is directly passed on to the user.

*MSW3*. Due to the importance of the mammalian supertree from [11] for phylotastic projects involving mammals, we implemented a new core service, called MSW3, based on taxonomic data from Mammal Species of the World, third edition [37]. The MSW3 taxonomy was downloaded as a CSV file from the MSW3 web site (see Table 1), and potential synonym names were extracted by searching for text beginning and ending with "<i>" and "</i>" tags in any column. A new CSV file consisting solely of these indexed names was stored as a local database. Three techniques are used to match names: (1) searching for an identical binomial name in the Genus and Species columns; (2) searching for names found anywhere in other columns in the CSV file, and (3) searching for names mentioned in different parts of a single row (e.g., the species epithet in the 'species' field, but the genus name mentioned in the 'Synonyms' column). These techniques identify all but a few percent of the 4500 names in the mammal supertree.

### Tree storage (TreeStore)

The focus of the TreeStore team was to develop a flexible way for phylogeny providers to expose knowledge for use in phylotastic systems.

#### Design considerations

A key feature of the overall architecture (above) is the capacity to choose a suitable tree from among available source trees, rather than being constrained to one or a few local or “built in” trees. The suitability of a source tree for a given case may be based not only on coverage of a set of species, but on metadata describing the methods, protocols, and data used to construct the tree (e.g., see [38]). Such metadata would include the recommended citation by which users can properly credit the source tree(s) in subsequent publications. Thus, a phylotastic tree-store should support flexible storing, querying, and retrieval of trees, and also of metadata associated with trees and their component nodes and branches.

#### Implementations

Standards and technologies for the semantic web [39] seem well positioned to address the nature of the functional requirements for the tree-store. In particular, we chose to use the Resource Description Framework (RDF) [40] to design a model for large phylogenies and their metadata; to use Virtuoso (OpenLink Software [41]) as a triple-store [42] that is scalable enough for storing very large phylogenies annotated with RDF; and to use Virtuoso’s implementation of SPARQL (the standard RDF query language) for querying the triple-store.

A critical step in enabling programmers and users alike to query the data in a triple-store is to design an RDF data model that, on the one hand, has the flexibility to accommodate diverse data, metadata and querying needs, and on the other hand, aligns well with controlled vocabularies and ontologies currently in use. We chose to use the Comparative Data Analysis Ontology (CDAO) [43]. We began by modifying CDAO to comply more fully with best practices in ontology engineering [44].

Designing the representation for the required metadata capabilities revealed several gaps in CDAO and other available vocabularies. As a consequence, we are building OWL ontologies and RDF models for TNRS matches of OTU labels, bibliographic citation of a tree, and for the various attributes encompassed by the proposed MIAPA reporting standard [38]. For example, the TNRS ontology (see Table 1) defines the classes and properties needed to represent the results from resolving OTU labels to taxonomic names.

### Pruning and grafting

#### Design considerations

The concepts of grafting and pruning are relatively simple, and could be implemented as part of a tree-store, but standalone services are part of the distributed design of a phylotastic system. While there was not a team dedicated to topology services, several hackathon participants developed proof-of-concept pruners, explored compute times for pruning, and refined existing pruning implementations.

#### Implementations

For small trees, pruning is already available for users of some phylogenetic programming platforms and software packages. However, for on-the-fly pruning of very large trees (e.g., 10^5^ taxa), the algorithms implemented in commonly used programming toolkits (e.g., DendroPy, [26]; Forester (see Table 1); NCL, [45]) may be too computationally intensive, especially if for each pruning step the tree structure is parsed out of parenthetical-formatted flat-text [46]. Preliminary tests confirm that this is the case, indicating that pruning using implementations such as DendroPy may take several minutes for a trees the size of the 55473-species tree of Smith, et al. [12]. Experiments with relational databases suggested that performing pruning operations using SQL might not yield satisfactory performance improvements either. In contrast, a prototype implemented using SPARQL and the Virtuoso triple-store suggested that such an approach could provide excellent performance.

Promising results were found using the MapReduce design pattern [47]. Our implementation, deployed on the development server with a convenient web-forms interface described below (see Table 1 for URI), suggested that pre-processing of the input trees into separate terminal taxon-to-root paths that are accessed in a parallelizable way could reduce processing times significantly (e.g., in the prototype implementation, shortening a pruning operation from ~20 minutes to ~6 seconds). Most of the performance gains of the prototype implementation are likely due to the pre-computed de-normalization of the tree structure into taxon-to-root paths. Because this implementation does not yet take advantage of parallelization, and requires the Hadoop MapReduce framework to boot up for each request, further performance gains may be anticipated.

Grafting and pruning are the core operations of Phylomatic [8], a pre-existing tool mentioned in Background. The online version of Phylomatic was upgraded (version 3) in connection with this hackathon (see Table 1 for URI), and is now implemented in gawk, a lightweight pattern-matching utility [48], drawing on no external libraries to parse Newick, NeXML or CDAO RDF phylogenies, and using simple node-to-parent-node arrays as its internal data representation. Pruning and grafting in Phylomatic are relatively efficient, requiring only 1.8 seconds to load a tree with 55000 tips and prune out all but 10 taxa (on an Intel i5 processor). Functionality that was added to version 3 includes: modifications to enable easier access and incorporation as a web-service; a range of built-in megatrees, not just for plants; and the ability to read and write NeXML and CDAO RDF, enabling the web-service to act as a format translator without any grafting or pruning.

### Scaling trees (Branch lengths)

The Branch Lengths group aimed to satisfy the user requirement for phylogenies that are not merely topological frameworks, but have branch lengths that reflect time or amount of divergence, based on incorporating relevant biological data.

#### Design considerations

Several possible automated approaches to scaling trees may be imagined, including sampling character data (e.g., sequences obtained from GenBank); assigning branch lengths by simple subdivision of root-to-tip path lengths into equidistant internodes (e.g., as in the bladj method from [8]) or using more sophisticated models of expected cladogenesis; calibrating the tree using fossil data; or combinations of these different approaches.

The Branch Lengths group opted to develop a system that assigns dates to nodes based on a stored library of fossil-calibrated trees (chronograms). This design was inspired by TimeTree [49], which takes a pair of species and returns point estimates of the age of their most recent common ancestor from published chronograms. TimeTree itself could not be used, as the terms of its license prohibit large-scale mining of its data, which are compiled from published work. Our initial design includes three elements: an input interface allowing the user to specify a list of taxa (two or more), a server that returns estimates of ages for most-recent-common-ancestors, and an interface to the results returned.

#### Implementations

Interaction with DateLife is primarily done through its website (see Table 1), though all the source code and data can be downloaded to run locally. The website is created using PHP, which also processes RESTful requests. This then calls an already-running R daemon, created using the FastRWeb [50] interface to RServe [51] as well as functions from ape [52] and new functions. This daemon returns the requested information as a JSON string, a Newick tree, or an HTML page. Internally the R scripts work as follows. Upon startup, input trees are pre-processed by converting them into patristic distance matrices for taxa. Then, satisfying a query by obtaining the ages for a set of taxa is simply a matter of matching row names and then subsetting the array to the relevant entries (rather than traversing a tree structure). Doing this for thousands of trees on a typical server takes under a second. The initial chronograms were placed in the PhyloOrchard R package as a temporary tree-store while others were being developed.

Importantly, rather than returning a single point estimate from a study the new tool allows a range of dates to be returned if there are multiple trees (such as post-burnin trees from a Bayesian analysis) from a study.

### Web site and special demonstrations (Shiny)

The goal of Team Shiny was to develop demonstrations showing the potential of phylotastic components, and to create a public face for the project.

#### Design considerations

The team considered demonstration projects that would be easy to understand, that would highlight the unique role of phylotastic systems, and that would relate to important research problems. The team sketched out five possible projects, prioritized as follows: (1) *Reconcili-o-tastic*; (2) a generalized phylotastic web interface; (3) *Mesquite-o-Tastic* (and other forms of integration with character analysis workflows); (4) *Phylo-Taxic*; and (5) *Phylotas-Doc*. The first 3 ideas are explained below; Phylo-Taxic would provide a phylogeny for a higher taxon such as a family or order; Phylotas-Doc would supply a phylogeny for the species named in a scientific paper or other document such as a web site.

#### Implementations

Team Shiny implemented 3 demonstration projects, deployed an informational web site (http://phylotastic.org), and produced a series of blogs and screencasts. While it was not possible to produce a fully generalized web interface (due to the lack of a fully implemented phylotastic system), the team built up the web interface to the MapReduce pruner (see Table 1) with explanations along with sample queries appropriate for each of the source trees.

Mesquite-o-tastic is a small demonstration of the utility of integrating phylotastic services into the kinds of workflows often used in evolutionary analysis, which are interactive and manually supervised workflows, often combining several separate pieces of software. Mesquite [53] is an extensible workbench for comparative evolutionary analysis written in Java, providing tools for uploading and manipulating lists of species (or other units of comparison such as genes or proteins), matrices of comparative “character data” and trees. We developed a small Java module that extracts a list of taxa from the data matrix loaded into Mesquite, and attempts to obtain a phylogeny for those species using the MapReduce pruner described above. Mesquite automatically integrates the tree with any character data, allowing phylogeny-based analyses of the character data, as shown in a screencast (see Table 1 and below, Results and Discussion).

The main product of Team Shiny is Reconcili-o-Tastic. As noted in the Background, reconciliation of gene trees with species trees [54] is potentially a high-volume use case for phylotastic services. Current reconciliation approaches assume that the user will supply a species tree. This requires the user to determine the set of species implicated by a gene or protein tree, then generate or otherwise obtain a tree for those species. In Reconcili-o-tastic, these steps are automated. In response to the choice of an initial gene tree (from among a set of examples provided), Reconcili-o-Tastic (1) reads the input tree; (2) queries external databases to link identifiers in the input file to species names; (3) uses these species names to retrieve the species tree phylotastically; and (4) performs reconciliation.

The operations are implemented as follows. Strings that match the pattern of gene identifiers are extracted from the input file using custom code. Sequence records are then retrieved from an appropriate database by invoking UniProt web services (see Table 1). Species names are obtained from these sequence records. Genes (proteins) for which a corresponding species name cannot be established, along with those missing from the phylotastic species tree, are removed from the gene tree prior to reconciliation. Reconciliation is done using a modified version of the speciation-duplication inference (SDI) algorithm described in [55], which allows for non-binary species trees. The result of this reconciliation is a gene tree with speciation or duplication events at each internal node.

Reconcili-o-Tastic is implemented as a web application in the web2py framework, using JavaScript for front-end operations, and drawing extensively for back-end operations on the Forester library (Java; see Table 1), which includes the SDI reconciliation engine and the Archaeopteryx viewer. Reconciled trees are represented (with encoded duplication and speciation nodes) in PhyloXML format [56]. Input gene trees, species trees, and reconciled trees are displayed interactively using Archaeopteryx [53], an embedded Java applet.

## Results and discussion

The aim of the phylotastic project is to develop a delivery system for expert knowledge of species phylogeny. In response to a user-supplied list of taxa, a phylotastic system identifies suitable source phylogenies, matches species identifiers, prunes away unneeded subtrees, grafts on missing species, and supplies branch lengths and other information, ultimately returning an expert phylogeny for the user’s list of species. Ideally such a system would cover all kingdoms of life, be fast enough to provide results while the user waits, and address the particular needs of researchers for reproducibility and provenance. To enhance the potential for such a system to become a sustainable community resource, it could be implemented as a set of loosely coupled components that interact in clearly defined ways, e.g., via web services.

### Steps toward enabling a phylotastic system

The implementations described above provide a point of reference for considering the potential of a phylotastic system as conceived here, and for identifying weaknesses. With these 2 goals in mind, below we discuss in particular, 3 demonstration projects: the MapReduce pruner, Mesquite-o-Tastic, and Reconcili-o-Tastic.

The MapReduce pruner can be invoked interactively via a convenient web form, which has a text box in which to enter a list of species, and pull-down menus to select a format (5 choices from Newick to NeXML) and a source tree (from a list that includes most of the large trees listed in Background). A tree is returned typically in 8 seconds. The web form is merely the front end to a web service that can be invoked via a URI with arguments for “species”, “tree” and “format”. A simple Perl script using this web-services API would be as follows:

This script could be invoked with a command such as

 and the result will be a file called “out.tre” with the Newick tree-string “((Homo_sapiens, Pan_troglodytes),Mus_musculus)”.

Demonstration software based on Mesquite [52], an extensible workbench, illustrates how such web services could be integrated into an interactive workflow. In the Mesquite-o-Tastic screencast (Table 1), a NEXUS file from a published scientific study [57] is downloaded from an online archive (MorphoBank [58]), and opened in Mesquite, which shows that the file contains a character matrix with 51 taxa, but not a phylogeny. When the user invokes a custom menu item (added for this project), Mesquite automatically formulates a query URI using the names in the character matrix, executes the query remotely (invoking the MapReduce pruner above) and incorporates the resulting tree in memory. The tree is then available for graphical display as well as for use in analyses such as reconstructing ancestral states. While we chose to create this demonstration using Mesquite, the same thing could be done with a variety of other software systems (e.g., DAMBE, PAUP, MEGA).

The Reconcili-o-Tastic demonstration shows how phylotastic services can be integrated into a more automated workflow, as described above (Shiny section). In this case, not only is the query constructed automatically (by retrieving species names from sequence identifiers), it is used automatically for a downstream analysis step, which is to generate a reconcile tree.

### Current limitations

Limitations of the tools described above become apparent quickly if one considers a broader set of cases than the sample queries used for illustration. Some of these limitations are due to limitations in the current state of expert knowledge, others are due to incomplete implementations of the phylotastic concept, and still others represent design limitations.

These limitations can be clarified relative to an imaginary challenge of (1) obtaining many sets of names, e.g., by downloading hundreds of NEXUS files from MorphoBank [58], or thousands of NEXUS files from TreeBASE [7], or processing thousands of scientific publications using GNRD (Table 1) to auto-recognize names, then (2) using the tools developed here to find phylogenies for implicated taxa, and (3) attempting to use the resulting phylogenies to carry out some kind of phylogeny-dependent downstream step, such as computing phylogenetic diversity, or reconstructing ancestral character states.

Current tools, if subjected to this kind of challenge, would prove unsatisfactory. The first challenge is that source trees available (via the MapReduce pruner) provide limited coverage of the millions of known biological species. The result is that, in many cases, only a minor fraction of species named in the query would be found in a source tree. This is largely a limitation in the coverage provided by available megatrees (the Open Tree Of Life project, mentioned further below, attempts to address this gap by synthesizing phylogenetic knowledge broadly). Coverage differs dramatically among different taxonomic groups, e.g., the mammal tree [11] covers the vast majority of extant mammals, but there is poor coverage of fungi and protists. Comparative studies of morphology often use fossil data, but fossil species are poorly represented in large phylogenies, because the latter typically are constructed from molecular sequences (typically not available for extinct species). Grafting of missing species onto the corresponding genus or family could improve coverage, but we have not implemented grafting methods here other than via web services supplied by the enhanced version of Phylomatic.

Currently available strategies for taxonomic name resolution also represent limitations. Our TNRS meta-service has not been integrated with other components, so that (for example) the names in a NEXUS file uploaded by Mesquite-o-Tastic must be spelled exactly as in a source tree available from the MapReduce pruner, or no match will be found. This may be a desirable behavior in some cases, e.g., when the user (or client application) is confident about names and does not want to allow any translation. However, in most cases, presumably, integrating the TNRS meta-service would improve results.

There are a number of current limitations on the potential for improvement, because (1) sources of taxonomic knowledge referenced by the meta-service (NCBI, MSW, etc.) provide limited coverage of names; (2) spell-checking typically is not available; (3) there is no automated way to choose among multiple matches (especially given the possibility of valid homonyms — identical names for different species covered by different nomenclatural codes); and (4) there is no automated way to interpret names from higher taxonomy. With regard to the last-named challenge, for instance, MorphoBank has many data matrices (e.g., project #816) that combine data at the genus level or higher, so that the key to a row of data is a higher taxon label (e.g., “Sabellidae”) or an anonymized species name (e.g., “*Myriowenia* sp.”). One can imagine a system that resolves such names in an appropriate way depending on the user’s choice, but support for such a system exists only for plant species and only up to the taxonomic rank of family [36].

The general design for a phylotastic system (given above) calls for a tree-store that responds to a user’s query by identifying a source tree that provides the best coverage. However, such a component has not been integrated, thus current tools require the user to specify a source tree in advance.

Integrating many source phylogenies, along with a TNRS and a tree-store, would make it possible to respond to a variety of phylotastic queries to identify the best source tree for each one. However, given our current implementations, the resulting trees would lack branch lengths and contain polytomies, making them unsuitable for many kinds of downstream analysis (for reasons noted earlier). The lack of branch lengths could be addressed by integrating the DateLife service, but currently its store of calibrated phylogenies covers only animals. This situation could be improved, but fossil-based calibrations generally are not available for phylogenies of groups of microscopic organisms, which have a poor fossil record. Other methods for scaling trees are possible (as noted above), but we have not explored those methods. Likewise, polytomies could be resolved arbitrarily, or using character-based methods, but we have not explored such options.

Finally, whereas we can imagine an enormously useful phylotastic system that efficiently delivers currently available knowledge of phylogeny, taxonomy, and fossil dating, current standards and technology for annotation are insufficient to enable the delivery of this knowledge with enough credibility for scientific research. Whereas students and educators may be satisfied to download a tree made by a “black box”, researchers will expect a clear description of sources and methods, including metadata on the source trees used to derive a result, and the phylotastic method of its derivation (by pruning, grafting, re-scaling, etc.). Yet standards for annotating sources and methods are relatively undeveloped (see discussion in [5]). Attribution and licensing present additional challenges for a system that re-uses data.

Beyond these narrow technical limitations there is the question of whether a fully developed phylotastic system ultimately would represent a practical alternative to other ways of obtaining phylogenetic information. The most obvious uses of such a system are for cases in which the user’s demand for speed is relatively high compared to the demand for rigor. Resources such as Wikipedia or the Encyclopedia of Life, for instance, might benefit from the ability to auto-generate phylogenies to illustrate a taxon for a taxon-specific web page. Whether scientists will use a phylotastic system for research purposes will depend on multiple factors that include the user’s demand for rigor, the user’s potential to infer— at a significant cost in terms of time, training, and computation— a more rigorous phylogeny by *de novo* methods, and the availability of a pre-computed expert phylogeny that covers the query of interest (and is considered authoritative).

Such factors are not easy to assess directly, but the examples cited by Stoltzfus et al. [5] suggest that, given the opportunity, researchers often will choose to forego the task of inferring a species phylogeny *de novo* from character data, and instead will choose to apply approximate methods to compute a derived tree from an expert source tree, even when the researcher’s needs are limited to a single tree with a few dozen species (e.g., as in [59]).

### A phylotastic ecology of informatics resources

The results described above provide a basis for further development of phylotastic systems in hackathons planned for the year 2013. This further development will take place within a community with a long history of cyber-infrastructure projects, the oldest being TreeBASE and ToLWeb, both of which date from the 1990s. More recent phylogeny-related resources include CIPRES [60] and PhyLoTA [61]. Taxonomic information services also have existed for many years (e.g., ITIS noted in Table 1). Our DateLife service is similar to the widely popular TimeTree project [49], noted above.

How does phylotastic relate to these other projects? How might the projects inter-relate in the future? Above (Implementation) we explained why we chose to implement a TNRS meta-service (rather than rely solely on an existing service), and why we chose to implement a new tree-scaling service similar to TimeTree. In both cases, the reasons relate to the need for resources that are designed (and licensed) to support automated data-integration tasks, rather than interactive or *ad hoc* uses.

Currently, although TreeBASE and ToLWeb are resources that represent expert knowledge of phylogeny, they are not alternatives to a phylotastic system as a convenient source of custom trees for downstream use. TreeBASE [7] provides tools for searching ~8000 published phylogenies— a small fraction of all published phylogenies [5]—, but it does not include pruning or grafting tools, nor does its store of trees include any of the trees given above (Background) as examples of large species trees.

ToLWeb is primarily an educational resource whose main feature is a phylogeny divided into branches curated by experts who determine the phylogeny and supply annotations. Its downloadable phylogeny of 16000 taxa covers all kingdoms but includes <1% of named species; as noted in [5], when bioinformatics researchers want a comprehensive ToL (e.g., for projects such as TimeTree), they use the NCBI taxonomy tree, which has 250000 species. Educational uses of ToLWeb predominate over research uses, perhaps because the interfaces focus on graphical presentation: when ToLWeb is cited in the research literature, in studies such as [62-67], it appears that knowledge of a small set of relationships is conveyed visually rather than by computation from the tree.

Resources such as PhyLoTA [61] and the CIPRES portal [60] clearly provide ways to generate custom species trees for downstream use. However, the trees are generated by the user *de novo* from sequence alignments. While implementing a phylogenetic inference workflow using CIPRES or PhyLoTA is far easier than implementing an ad hoc workflow on a local computer, it is time-consuming and represents a substantial burden for most users.

By contrast, the phylotastic project aims to facilitate the case in which a user can make a scientifically defensible choice to use a modified (pruned, grafted, re-scaled) version of an expert phylogeny, rather than attempt to infer a phylogeny *de novo*. Clearly Phylomatic [8]—the inspiration for the phylotastic project—also addresses this niche. While the original conception of Phylomatic was a local tool with a fixed source tree, subsequent developments (including developments for this project) expanded its web-services interface and allowed the capacity for a user-supplied tree, allowing Phylomatic to become a component in a distributed phylotastic system of components that interoperate to provide on-the-fly access to ever-expanding domains of expert knowledge (of phylogeny, taxonomy, geographic distribution, etc.).

Just as Phylomatic was designed for a smaller and more static world of data, but has begun to adapt to a larger and dynamic world, the other resources listed above also could play a role in this emerging world. As described above (Implementation), existing taxonomic name resolution services can be adapted for aggregation into a meta-service. Likewise, existing phylogeny resources such as ToLWeb or TreeBASE could expose their content using the TreeStore concept envisioned in Figure 2. For instance, for this project, we exported the XML version of the ToLWeb tree, and translated it using Bio::Phylo [25], so that the content of the ToLWeb tree is available via the pruner web tool described above. If ToLWeb were to supply the current version of its tree via a web service, this could be accessed by the pruner; likewise, TreeBASE could expand its current web-services interface to expose its data to phylotastic systems.

The goal of the phylotastic project is to leverage expert knowledge of phylogeny, rather than create *de novo* trees using tools such as PhyLoTA and CIPRES. Yet, there are cases in which a phylotastic system could benefit from rapid methods for making limited phylogenetic inferences from a sample of sequences or other data, including (1) using phylogenetic placement [68] to place a missing species on a tree, when taxonomic grafting is impossible or undesirable, (2) resolving a polytomy, or (3) assigning branch lengths within subtrees of organisms poorly represented in the fossil record (e.g., single-celled organisms).

The design of phylotastic systems allows for perpetual heterogeneity and novelty, thus it does not matter whether or not a central source of authoritative ToL knowledge emerges in the next decade through efforts such as the Open Tree Of Life project (Table 1). New phylogenies will augment available tree-stores, and phylotastic systems will allow them to be pruned, grafted and analyzed according to the wishes and needs of the user. Because of the open architecture and modularity of the project, researchers can chain the phylotastic components together in various ways, piecemeal or as complete workflows.

Finally, if successful, a convenient and comprehensive delivery system for expert phylogenetic knowledge will create a competitive marketplace in which alternative source trees, and alternative phylotastic services, compete to satisfy the demands of users. The existence of such a marketplace may be expected to catalyze broad improvements in related technology and standards. For instance, given that the scale of scientific phylogeny re-use has been— with the exception of APG and Phylomatic— unimpressive [5], the delay in developing a “minimal information” standard for annotating phylogenies, first proposed in 2006 [38], is unsurprising. However, a phylotastic system will require annotations of sources and methods to satisfy the demand of researchers for credible (publishable) results and, given the choice, users will prefer those source trees, tree-stores, and client applications that provide them with more fully annotated results. Another critical feature missing from the technology landscape of phylogenetics is some scheme for quantifying the accuracy or perceived quality of phylogenies— e.g., an objective scheme based on consistency or metadata density, or a subjective scheme based on social bookmarking—, but one can expect such a scheme to emerge naturally as an aid to users facing choices in a phylotastic marketplace.

## Conclusions

The expanding scope of available species phylogenies, and the increasing demand for custom phylogenies for use in evolutionary analysis, suggests the value of a generalized phylotastic system that, in response to a user’s query consisting of a list of names, would provide name-resolution, tree-finding, pruning, grafting, scaling, and annotation operations necessary to generate a custom phylogeny for the named entities. Approximately 9 person-months of effort were devoted to a carefully planned hackathon at which 2 dozen participants worked to develop such a system. The results of this hackathon demonstrate the feasibility of some aspects of the project, such as rapid pruning and re-scaling, and expose remaining challenges, such as providing an integrated spell-checking system for mapping input names to qualified taxonomic identifiers. The project has demonstrated the feasibility of on-the-fly delivery of expert phylogenetic knowledge under limited conditions. Further work is needed to develop a production system that is robust and scalable, and which can be adapted to multiple use-cases. If such a system can be developed, it may be expected to drive improvements in other areas of the worldwide effort to assemble a ToL.

## Availability and requirements

**Project name:** Phylotastic;

**Project home page:**http://www.phylotastic.org;

**Operating systems:** Linux, MacOSX;

**Programming languages:** Perl, Java, R, Python, JavaScript, PHP, SPARQL, awk;

**Other requirements:** as described for individual sub-projects;

**Licenses:** GPL3, MIT, BSD 3-clause;

**Any restrictions to use by non-academics:** No.

The project website (Table 1) describes the phylotastic project and provides links to demonstration software (“demos”), web services produced during the hackathon, the working project wiki, and code hosted on GitHub. The screencasts (see Table 1) are available on YouTube and are readily discovered by searching with the keyword “phylotastic”. The requirements differ for the different software products. These products generally are free of dependencies on commercial software.

Source code for most products is available at GitHub under an open-source license, with the following project names: phylotastic/tnrastic (TNRS metaservice); phylotastic/tolomatic (MapReduce pruner); phylotastic/arch-galaxy (Galaxy tool integration); helenadeus/phylotastic_js (javascript controller); phylotastic/cgi (CGI controller); phylotastic/mesquite-o-tastic (Mesquite-o-Tastic); phylotastic/phyloShiny (Reconcili-o-Tastic); phylotastic.github.com (project web site); camwebb/phylomatic-ws (Phylomatic 3). The DateLife web site (Table 1) includes links to its source code (currently on BitBucket with the identifier bomeara/datelife).

Access to live demonstrations and documentation is as follows. The MapReduce pruner is accessible via a convenient web-forms interface that provides instructions and examples (see Table 1). Reconcili-o-Tastic is implemented on the NESCent development server (Table 1) and may be installed locally (using web2py) following the instructions in the README file on GitHub. Mesquite-o-Tastic is not implemented on the server, but instead should be evaluated locally: the code available on GitHub (above) may be added to a local installation of Mesquite simply by copying the code into the proper local directory, as explained in the generic instructions for installing modules provided on the Mesquite project web site (Table 1). The Mesquite-o-Tastic screencast (Table 1) serves as the documentation. The DateLife presentation at the 2012 iEvoBio conference (Table 1) serves as the documentation for DateLife.

Finally, because some products may require special expertise to install, we have created a stand-alone server image that can be mounted by a computer system administrator without expertise in bioinformatics (see Table 1). A server image is a snapshot of an operating system disk that can be started up inside a host environment as a virtual machine (VM). This server image includes all of the principal working products of the hackathon, including those listed above, as well as the Virtuoso tree-store with a web-services interface, and excluding only DateLife, Phylomatic 3, and Mesquite-o-Tastic.

## Abbreviations

APG: Angiosperm Phylogeny Group; API: Applications Programming Interface; CDAO: Comparative Data Analysis Ontology; CGI: Common Gateway Interface; HIP: Hackathons, Interoperability, Phylogenies; ITIS: Integrated Taxonomic Information Service; JSON: JavaScript Object Notation); MIAPA: Minimum Information About a Phylogenetic Analysis; NCBI: National Center for Biotechnology Information; NCL: NEXUS Class Library; NESCent: National Evolutionary Synthesis Center; LT: Leadership Team of HIP; MSW3: Mammal Species of the World, version 3; OBO: Open Biomedical Ontologies; OTU: Operational Taxonomic Unit; PDF: Portable Document Format; rDNA: Ribosomal DNA (deoxyribonucleic acid); RDF: Resource Description Framework; REST: Representational State Transfer; TNRS: Taxonomic Name Resolution Service; URI: Uniform Resource Identifier; US: United States

## Competing interests

The authors declare that they have no competing financial interests. Some authors are key participants in projects mentioned in the text, including NeXML (RV, MH, JS, PM, JB, A Stoltzfus), CDAO (JB, EP, A Stoltzfus, HL), TreeBASE (RV), Mesquite (PM), MIAPA (KC, HL, A Stoltzfus, JB, RV, EP) and the iPlant TNRS (NM).

## Authors’ contributions

Authorship on this work was open to all who participated in the hackathon or its leadership team. The order of authors reflects post-hackathon contributions to the manuscript and to hackathon products. The HIP Leadership Team (AS, BS, EP, HL, KC, MR, MW, RV, SP and MW) conceived and planned the hackathon, with administrative coordination by AS, assisted by RV and EP. All authors except BS, MR, and MW participated in the hackathon, with BO, LH, JB, MWP, and MA doing so remotely. With the exception that PM wrote the Mesquite code for Mesquite-o-Tastic, and GJ extensively revised Reconciliotastic for purposes of this publication, the hackathon teams are responsible for products attributed to them above: DateLife (BO, TH, PM, LH, MWP); TNRS (NM, GV, SM); Shiny (CZ, HB, MP, AS); Architecture (HD, RV, COW, JS), including Pruners (RV, A Steele, COW); TreeStore (HL, KC, JPB, EP). Most of the authors contributed to initial drafts of Implementations; initial drafts of the Background and Discussion were written by AS with help from BS; revisions were done primarily by AS, with help from HL, HD, BS and EP. AS coordinated work on the manuscript. All authors read and approved the final manuscript.
